# Funders' Perspectives on Supporting Implementation Research in Low- and Middle-Income Countries

**DOI:** 10.9745/GHSP-D-21-00497

**Published:** 2022-04-28

**Authors:** Ana Cardoso-Weinberg, Chris Alley, Linda E. Kupfer, Garry Aslanyan, Michael Makanga, Fabio Zicker, Ole F. Olesen

**Affiliations:** a European & Developing Countries Clinical Trials Partnership, EDCTP, The Hague, The Netherlands.; b Hunter College, City University of New York, New York, NY, USA.; c Fogarty International Center - National Institutes of Health (NIH), Bethesda, MD, USA.; d TDR, the UNICEF/UNDP/World Bank/WHO Special Programme for Research and Training in Tropical Diseases, Geneva, Switzerland.; e European & Developing Countries Clinical Trials Partnership, EDCTP, The Hague, Netherlands.; f Fundação Oswaldo Cruz (Fiocruz), Rio de Janeiro, Brazil.; g European Vaccine Initiative (EVI), Heidelberg, Germany & Global Health Section, Department of Public Health, University of Copenhagen, Denmark.

## Abstract

We identify and discuss 7 approaches for funders to contemplate when considering support for implementation research in low- and middle-income countries.

## Key Messages

- Implementation research (IR) is an emerging research area that helps research and public health programs achieve impact at scale. We sought to collate and share health research funders' experiences with funding of IR activities in low- and middle-income countries (LMICs).
- We identified and grouped the funders' strategies into 7 approaches that are considered important when supporting IR in LMICs.
- Our analysis offers broad direction and guidance to funding agencies and related partner organizations on important elements to consider when funding and implementing IR in LMICs. Ultimately, this work could help improve the impact of research investments in LMICs.

## BACKGROUND

Odeny et al. identified 73 unique definitions of implementation science.^1^ However,

*almost all definitions of implementation science and research referred explicitly or implicitly to the gap between knowledge and evidence from research findings (i.e., something theoretical) and use, delivery, and application (i.e., something that is actually done).*

Reflecting ongoing debates about definitions, we found funders of implementation research (IR) to be hesitant to rely on any single definition of implementation science. Therefore, we do not suggest that funders of implementation science have exclusively used any single definition but rather seek to convey an appreciation of the significant breadth of definitions that exist. That said, for clarity, in this commentary, IR is understood to be a field of science that helps to ensure research findings are adopted into practice.^1^

Implementing research findings into practice is necessary to achieve a return on research investments and improve health outcomes in the broader community.^2^ Nevertheless, a chasm often remains between the development of efficacious health interventions and their optimum delivery in real-life settings. This gap is particularly present in many low- and middle-income countries (LMICs) where, for example, massive amounts of routine data are collected within public health systems such as by ministries of health and nongovernmental organizations, but are underused, reducing the potential effect of research on policy and practice.^3^ Funders committed to improving health in LMICs are now increasingly investing in IR. These funders have developed different strategies to support IR and in doing so facilitate the implementation of research findings in various ways.^4^ By investing in this field, funders aim to understand and contribute to bridging the gap between “knowing and doing.”^5^ Better understanding of how to bridge this gap may encourage funders and research institutions to leverage past, present, and future investments in health research, and thereby better contribute to improving health outcomes throughout the world.^6^

By investing in implementation research, funders aim to understand and contribute to bridging the gap between “knowing and doing.”

Recent years have seen a rapid and continuing development of implementation science as a field.^4^ The sharing of information to better understand this new area of research and how best to fund it is, therefore, urgently needed.

Health research funders are in a unique position to support the translation of research findings into health policy and practice,^7^ as they are often key catalysts of and contributors to science and policy integration.^8^ ESSENCE (Enhancing Support for Strengthening the Effectiveness of National Capacity Efforts) on Health Research is an initiative that coordinates and harmonizes funders' investments in health research capacity in LMICs. Funding agencies that are members of ESSENCE formed a Working Group on Implementation Science in 2017. The working group prioritized the following areas requiring further elucidation: an understanding of how the broader health research funding community has been supporting this area, the opportunities or challenges in supporting IR from a funder's perspective, and recommendations on how to successfully invest in and manage this research area.

We aim to present the views of 20 health research funding agencies on the best ways to invest in IR in LMICs.

First, ESSENCE on Health Research members participated in a survey to identify which funding agencies were currently supporting or interested in future support for IR in LMICs; map IR programs and activities currently being supported; and understand what funding agencies perceived to be necessary to inform their current and/or future investments in IR. For the key informant interviews, we purposefully selected funding agencies that were members of ESSENCE and that had responded to the survey. Then, we expanded to funders outside of ESSENCE based on a desk review and through snowball sampling.^9^ Agencies included in the sample either supported IR at the time the study was conducted or wanted to support IR in the future. This yielded insight into these agencies' first-hand experiences, as well as those of agencies that did not have experience at the time of the interview but that were considering entering the field (Box). All selected funding agencies reported engagement in supporting a broad range of research projects and initiatives internationally, both in communicable and noncommunicable diseases, in research institutions and public health programs, mostly in LMICs. The Supplement contains additional details on the potential limitations of the sample of funders.

BOX **Funding Agencies Interviewed on Supporting Implementation Research in Low- and Middle-Income Countries**

**Privately Funded Institutions, International Focus**

Bill & Melinda Gates Foundation

Doris Duke Charitable Foundations

Institute Pasteur

Wellcome Trust

**Multiple Country, Government-Funded Institutions, International Focus**

European & Developing Countries Clinical Trials Partnership

Gavi, the Vaccine Alliance

Global Alliance for Chronic Diseases

TDR, the UNICEF/UNDP/World Bank/WHO Special Programme for Research and Training in Tropical Diseases

WHO Global Malaria Programme

World Bank

**Single Country, Government-Funded Institutions, International Focus**

Swedish International Development Cooperation Agency

International Development Research Centre, Canada

United States Agency for International Development

**Single Country, Government-Funded Institutions, National Focus (but support international research)**

Fundação Oswaldo Cruz–Fiocruz, Brazil

Research Council of Norway

Instituto de Salud Carlos III, Spain

Medical Research Council, United Kingdom

U.S. National Institutes of Health, The National Heart, Lung, and Blood Institute

U.S. National Institutes of Health, Fogarty International Centre

U.S. National Institutes of Health, National Cancer Institute

We developed an interview protocol (Supplement) from the survey results. The interviewers explored funding approaches, strategies, and enabling factors and barriers for success. They also collected data for case studies, success stories, failures, and recommendations, as well as strategic and operational information from the 20 agencies. Questions about investing in IR in LMICs from funders intending to do so in the future were also explored.

Between May and September 2019, we conducted a total of 28 semistructured interviews with staff members from the 20 selected health research funding institutions. Interviewees included staff members responsible for IR programs and members of management teams responsible for the strategic, scientific, and funding planning for their organization. Interviews were conducted by telephone with follow-up by email to address outstanding issues when necessary. Notes were taken from the interviews, which were then analyzed thematically. The findings and discussions were consolidated into key selected themes. We identified and reviewed patterns and themes from the perspective of the participants and verified and refined the findings through stakeholder discussions facilitated by ESSENCE.

### Ethics Approval

Human subjects research ethics approval to conduct interviews was given by the Research Ethics Committee for Science and Health at the University of Copenhagen. Ref: 504-0075/19-5000.

## FINDINGS AND DISCUSSION

From the interviews, we identified and grouped the funding agencies' strategies into the following 7 approaches (not in order of importance) that are considered important when supporting IR in LMICs: (1) involve all relevant stakeholders from the outset and throughout the entire research and delivery process; (2) embrace and leverage the diversity of IR funders; (3) increase awareness and readiness to implement new IR knowledge; (4) consider partnership building as central to IR, especially at the start; (5) promote prioritization of capacity building for IR within funding agencies and among wider audiences; (6) create and communicate clear funding criteria for IR; and (7) address sustainability by ensuring that IR skills and knowledge are embedded in national academic and health systems.

### Involve All Relevant Stakeholders From the Outset and Throughout the Entire Research and Delivery Process

IR is most effective and sustainable if end users—health practitioners, patients, health administrators, policy makers, funders, and other community stakeholders—are actively involved not only from the inception of but also throughout the research process. This requires consulting these various groups in open and transparent ways and asking them to help identify specific research needs and concerns. Equally crucial is informing them about the research process and how and when they can contribute most effectively. These consultations were considered a priority for respondents as they form a solid basis for the implementation of research findings and increase the chances of research outcomes addressing the needs of the communities involved. Involving all stakeholders from the start and throughout the process can help ensure this engagement is at the forefront of the project.

Involving all stakeholders from the start and throughout the process can help ensure this engagement is at the forefront of the project.

Funding agencies and the broader health research community are increasingly aware of the importance and complexity of broad stakeholder involvement in ensuring that investments made in research programs have a wide impact.^10^^,^^11^ They are in a unique position to support the collaborative process of research codesign and meaningful end user engagement across all stages of the research process.^12^^,^^13^ Funders can contribute to this process in various ways: ensure sufficient time for consultations with end users as part of their funding strategy, fund systems and platforms focusing on addressing this gap, and fund education and training of stakeholders as part of their effective participation in the IR process.^14^ Publishers can also mandate that researchers report on stakeholder involvement for IR projects about which they publish.

Funding agencies can consider financing the formation of new IR platforms to facilitate the wider study and documentation of end users' perspectives, which will, in turn, lead to research that is increasingly comprehensive, relevant, and tangible. Our analysis indicates that a best practice for funders could be to require that stakeholders are involved from the start and throughout the project and that their involvement is a core aspect of all IR proposals and reports. Indeed, as Sturke et al. observe^15^:

*Enhanced interactions between researchers and users of scientific evidence are important and necessary to tackle enduring barriers to implementation.*

An interview respondent stated:

*Sustainability of interventions means you need to begin with the end in mind. The right stakeholders must be engaged from the beginning to scale up interventions. This is what funders need to do, ensure that their grants have the applicant think about sustainability from the get-go, including having requirements that relate to having people that will be sustaining the intervention as part of the project team.*

Acknowledging that priority setting for investments in research is often determined without input from all relevant stakeholders, and that this results in a gap between evidence needed and evidence produced, the U.S. Agency for International Development (USAID) has funded health evaluation and applied research projects through a consultative approach. This was^16^:

*rooted in an understanding that the use of evidence to improve health policies and programs requires the long-term active engagement of multiple actors representing an extensive array of skill sets and experiences*.

USAID's consultative process has actively engaged funders, researchers and academic institutions, health practitioners, policy makers, and community advocates at the global, regional, national, and subnational levels across Asia, Africa, Europe, and North America. IR priorities pursued under this approach are iteratively developed through a combination of approaches, including literature reviews, rapid scoping reviews, interviews, stakeholder meetings, and surveys. By using multiple means to solicit and confirm priorities, USAID has been able to generate robust IR agendas on a range of topics. While it might not be possible to fund the full IR agenda immediately, the process helps to prioritize IR in ways that are relevant beyond specific funding opportunities and ensures that important issues are followed up in the future.

### Embrace and Leverage the Diversity of IR Funders

IR funders have relatively diverse profiles in terms of thematic focus, grant holders, funding rules, and amount and duration of funding, as well as monitoring and evaluation strategies.

Some funding agencies have a specific mandate and dedicated programs to support IR (e.g., U.S. National Institutes of Health [NIH]) while others function in more ad hoc or opportunistic ways and have, in some cases, embedded IR as part of larger funding programs (e.g., clinical trials). This can be a practical way for funders to gain experience with this kind of research, and thereby help to formalize an organization's long-term commitment. According to respondents, it has taken some time for health research funders to see IR as critical to their mission.

Our analysis indicates that, ideally, the importance of IR could be reflected in the long-term strategic thinking and planning done by funders. In the meantime, several agencies have looked for opportunities to “learn by doing” within the scope of their existing mandate, thereby gaining experience in investing in IR, honing their expertise, and helping to inform the design of well-conceived and appropriate approaches to this important issue. In some cases, funding agencies form alliances to coordinate funding or share best practices on IR funding and management. One such example is the ESSENCE Working Group on Implementation Research in which 13 funding agencies participate to share experiences and align strategic agendas. Related to this, an interview respondent said:

*I am not sure there is a model way to fund IR. While we use fairly standardized processes, our examples might not work for others that have their own policies and requirements. But there are great examples of how major funders across the world get together to fund and increase investment in a particular area. We shouldn't think about what model the individual funder uses but rather about a larger model that allows using one's own funding mechanisms to reach a common goal.*

### Increase Awareness and Readiness to Implement New Knowledge in IR

Funders have remained alert and responsive to the challenges and opportunities that IR offers as it evolves. A few respondents said that the potential for improving health and health care should motivate funders to overcome its baffling diversity of meanings and processes and that the definitional variability that exists may offer opportunities to funders and beneficiaries alike. One of the respondents proposed that, ultimately, the goal of IR is to create comprehensive programs that will help transform individuals' health. Other interviewees considered that implementation has happened when the “real world,” national approval, or national policy has changed. They also considered that some global IR leaders and experts can help shape language that appeals to both a broader audience and implementation scientists so that it can be as inclusive as possible.

New definitions, methodologies, theoretical approaches, frameworks, and models are continually emerging in IR. Some funders have supported this fast-paced environment by financing efforts to develop new theories and innovative methodologies built through multidisciplinary and multisectoral collaborations that have the potential to vastly improve health outcomes in LMICs. As an illustration of how rapidly the field of IR is gaining acceptance and how collaboratively it can operate, we note that in 2002, the U.S. National Institute of Mental Health alone issued its first funding opportunity announcement (FOA) for dissemination and implementation research in mental health. In contrast, by 2019, 21 of 27 NIH institutes, centers, and offices were part of the trans-NIH FOA for dissemination and implementation research in health. The 2019 FOA, like those before it, called for studies to advance IR methods, measures, and research dissemination. Among many other areas of IR, the current FOA allows for studies that focus on testing theories, models, and frameworks for dissemination and implementation; developing valid and reliable dissemination and implementation outcome and process measures; and developing study designs, research methods, and analytic approaches for studying dissemination and implementation.

Some funders have supported this fast-paced environment by financing efforts to develop new theories and innovative methodologies that have the potential to vastly improve health outcomes in LMICs.

### Consider Partnership Building as Central to IR

From our discussions, we noted that funding agencies use different partnering approaches and various types of creative platforms that help move the field forward. Funders generally work together because they share strategic goals, such as improving the quality of health care in resource-constrained countries (Table). By pooling their talents and resources, funders increase the likelihood that proven strategies, clinical interventions, and policies will make an impact beyond discovery research and create sustainable improvements in the health of populations. International partnerships were found to be ways to work around particular challenges and share accountability. In some cases, funding agencies partner to jointly allocate funding to an entity that runs a program for them, as was the case of the small grants scheme for IR on infectious diseases of poverty that was a joint initiative between the World Health Organization Regional Office for Africa; TDR, Special Programme for Research and Training in Tropical Diseases; and the European & Developing Countries Clinical Trials Partnership. In many cases, members of international alliances are encouraged to align research agendas and support joint programs, for example, as facilitated by the Global Alliance for Chronic Diseases.

**TABLE.** Goals and Benefits of Partnerships That Invest in Implementation Research in LMICs

GOALS	BENEFITS
Use assets and programs to achieve the same strategic goals.	By aligning their resources and programs, funders increase the likelihood that proven strategies and clinical interventions are put into practice and adopted into policy in country.
Jointly address challenges and share risks.	Through partnerships, challenges can be faced together, and risks can be reduced. One partner can run the program while another can contribute funding.
Support national partnerships and platforms that can promote implementation research training and mentorship, research opportunities, and stakeholder involvement.	Create a forum for national researchers and stakeholders to participate in implementation research and training and form national partnerships.

At the national level, funders, including country government funders, can invest in partnerships that create IR training opportunities and IR mentorships. These national partnerships not only have the benefit of identifying IR activities within a single country but also create a forum for national stakeholders to come together and share information. National partnerships that receive support from external funders also make it easier for funders to recognize and invest in places where promising research is being done. A new mechanism proposed by ESSENCE in 2019 facilitates the identification of gaps in capacity in LMICs and provides opportunities for relevant stakeholders to work together to address these gaps.^17^

### Promote Prioritization of Capacity Building for IR Within Funding Agencies and Among Wider Audiences

New ways of building capacity in IR are urgently needed, and this was raised at different levels during our discussions with funding agencies. As a relatively young field, IR has few bona fide experts and, consequently, relatively few mentors who can support students and early-career scientists. In LMICs, the shortage of researchers with IR experience and of institutions with the expertise to develop and run IR training programs is restricting the development of the field.^18^ As respondents said:

*There are few applicants right now… it feels like the same applicants, we need to find ways to get new people on board.*

*We are lacking rigor and a real knowledge of the methodologies as there is the idea that everybody can be an implementation researcher.*

Researchers and research offices within research institutions can be trained in IR, and funders can play a role in nurturing and sustaining researchers' commitment to learning about IR through awarded grants. It is crucial to create a professional environment for this work and a career progression through it. Funders can support this career progression through fellowship programs that fund early- to mid-career and senior scientists. Funders can also support the research community to respond to a request for proposals, for example, through courses explaining what IR is (e.g., such as the massive open online course organized by TDR, Special Programme for Research and Training in Tropical Diseases) and grant proposal writing (e.g., as organized by the European & Developing Countries Clinical Trials Partnership). Funders can also make resources available to guide the research community (such as the Global Alliance for Chronic Diseases Researcher Resources directory^19^). These efforts are particularly important in LMICs, where region-specific constraints play a major role and few applicants have opportunities to obtain formal training or mentorship in proposal writing. However, as an interview respondent observed:

*Just training individuals won't strengthen the health system. What must be done is simultaneously support a large-scale national intervention, so everyone has a shared vision or shared goal to achieve. And then within that, supporting implementation research that leads to solving problems that relate to that end goal.*

Funders can play a role in nurturing and sustaining researchers' commitment to learning about IR through awarded grants.

Another respondent said:

*We have to build standards, quality, careers, and education, collectively. It's no good just getting people to be able to write a grant, we need to get a global recognition that this kind of science can really affect what's happening in the country, and for this you need people within the health systems of these countries to get training to recognize the value.*

The value of cross-disciplinary teams to help expand the pool of knowledge, skills, and resources that IR programs can draw on was also raised. At each stage, efforts can be made to improve the abilities of all stakeholders to understand and use the knowledge generated by IR. This also includes building capacity within funding agencies to ensure that IR is built into strategic planning and all stages of grant evaluation and management.

### Create and Communicate Clear Evaluation Criteria

It is helpful for funders to clearly communicate the specifications and criteria they use to score, evaluate, and assess proposals or applications, which can vary among funders. Also, many grant applicants have limited training or experience in writing strong funding proposals, partly because this is a relatively new field. When funding cross-regional and global research consortia, funders can encourage and enable researchers in LMICs to participate as equal partners in proposal design and specification while clearly stipulating what a robust, responsive grant proposal should include. It has been proposed that funding agencies write funding announcements using the concepts and terminology that they would like to find in the research proposals that are submitted. The use of clear criteria is also important to help funders evaluate proposals and to brief external reviewers when needed.

### Ensure That IR Outcomes and Skills Are Integrated Into National Health Systems

For IR investments to be worth the resources invested, outcomes should be envisioned and designed to become an integral part of health and other social systems, which is a key desirable outcome of the funding. Agencies can contribute to this by ensuring that researchers' and practitioners' engagements with health policy and related realms are a key component of IR-related investments in LMICs. Reliance on external sources of funding means that there is a risk that IR outcomes will not be embedded in national and local health systems. Answers to IR questions are highly specific to each context, and they differ within countries and subnational regions, but if IR skills are embedded into the system itself, it becomes possible to connect the local to national and even global networks. The need for the ability to integrate outcomes and skills into the national health system has been made evident with the ongoing coronavirus disease (COVID-19) pandemic, which demonstrated that in addition to providing access to vaccines, therapies, and tests, countries need sufficient infrastructure such as laboratories, technicians, and cold storage, all of which can vary greatly among subregions.^20^ Also needed is the ability to disseminate information about prevention and understand how to implement effective preventative measures (for instance, the wearing of masks and the ability to address issues such as mask and vaccine hesitancy). An example of how such integration is encouraged is the SORT IT model (Structured Operational Research and Training IniTiative) coordinated by TDR, Special Programme for Research and Training in Tropical Diseases, which supports countries and institutions to conduct operational/IR around their own priorities.^21^

Funding agencies can contribute to integrating outcomes into the health system by ensuring that researchers' and practitioners' engagements with health policy are a key component of IR-related investments in LMICs.

Funding agencies can contribute to this integration effort in several ways: (1) prioritize support for projects that are designed to take IR skills into the overlapping streams of activity that together constitute health systems; (2) support programs that identify underlying factors that point the way toward the establishment and expansion of networks that foster the link between researchers, policy makers, and implementers, as well as ensuring that these relationships are always open and transparent and that those involved remain accountable to the citizens they serve; and (3) fund research on how IR can influence health policy as part of the broader process of policy dissemination and IR.

In addition, funders could consider supporting efforts for “de-implementation,” which is the discontinuation of the use of a health service or practice that is not effective, proven, harmful, or inappropriate. Some funders, such as the U.S. National Cancer Institute and NIH, have already begun to examine processes for de-implementation research, which shows the importance of integrating IR into health systems. A final option for ensuring cost efficiency and integration would be embedding research into national and academic health programs and health systems in LMICs. In this way, the traditional calls for funding applications launched by funding agencies could be complemented with funding made available within the local program structure.

## CONCLUSIONS

Funding agencies are becoming increasingly aware of the importance of health research uptake into policy and practice. Based on our analysis, an ever-increasing number of funding agencies are progressively supporting the development and use of frameworks and methodologies that underpin IR. They consider IR an important way of ensuring that investments in research are translated into action. This contrasts with what often occurs with discovery science which, when not supported through the continuum of science, including IR, has little hope of achieving health system impacts. Through IR, there can be alignment between efficacious research findings, policy changes, and improvements in the population health of a country. While not all funders have grasped the advantages of prioritizing and investing in IR, many have asked how they can best support this new field. Although there is increased interest and growth in the field, there are not enough IR experts in some funding agencies to allow them to feel comfortable investing.^10^^,^^11^

Our analysis offers broad direction and guidance to funding agencies and related partner organizations on important elements to consider when funding and implementing IR in LMICs. We hope that sharing funders' and managers' perspectives will provide clarity on the factors and processes that facilitate funding of robust IR programs and projects, which can ultimately be useful for the research community. The 7 key approaches cover issues related to engaging stakeholders, developing partnerships, and embedding IR capacity within both funding agencies and health systems. Moreover, the approaches will encourage an organization just entering the field of IR to find partners to work alongside. The analysis points to opportunities for shaping a global research agenda for IR. Ultimately, this commentary aims to help improve the quality and impact of future IR investments and to improve the health of populations worldwide.

## Supplementary Material

GHSP-D-21-00497-supplement.pdf

## Notes

**Peer Reviewed**

**First published online:** March 18, 2022.

## References

[1] Odeny TA Padian N Doherty MC. Definitions of implementation science in HIV/AIDS. Lancet HIV. 2015;2(5):e178–e180. 10.1016/S2352-3018(15)00061-2. 26423000

[2] Brownson RC Colditz GA Proctor EK, eds. Dissemination and Implementation Research in Health: Translating Science to Practice. Oxford Press; 2012.

[3] Quaglio GL Ramsay A Harries AD. Calling on Europe to support operational research in low-income and middle-income countries. Lancet Glob Health. 2014; 2(6):e308-310. 10.1016/s2214-109x(14)70218-9. 25103290

[4] Brantnell A Baraldi E van Achterberg T. An inductive exploration of the implementation knowledge of research funders. Health Res Policy Syst. 2019; 17(1):67. 10.1186/s12961-019-0472-8. 31319867 PMC6637601

[5] Graham ID Kothari A McCutcheon C; Integrated Knowledge Translation Research Network Project Leads. Moving knowledge into action for more effective practice, programmes and policy: protocol for a research programme on integrated knowledge translation. Implement Sci. 2018;13(1):22. 10.1186/s13012-017-0700-y. 29394932 PMC5797415

[6] Peters DH Tran NT Adam T. Implementation Research in Health: A Practical Guide. Alliance for Health Policy and Systems Research, World Health Organization; 2013. Accessed February 28, 2022. https://www.who.int/alliance-hpsr/resources/implementationresearchguide/en/

[7] Holmes B Scarrow G Schellenberg M. Translating evidence into practice: the role of health research funders. Implement Sci. 2012;7(1):39. 10.1186/1748-5908-7-39. 22531033 PMC3420241

[8] Smits PA Denis JL. How research funding agencies support science integration into policy and practice: an international overview. Implement Sci. 2014;9(1):28. 10.1186/1748-5908-9-28. 24565209 PMC3939639

[9] Heckathorn DD. Respondent-driven sampling: a new approach to the study of hidden populations. Soc Probl. 1997;44(2):174–199. 10.2307/3096941

[10] Alonge O Rodriguez DC Reveiz L Peters DH Brandes N Geng E. How is implementation research applied to advance health in low- income and middle-income countries? BMJ Glob Health. 2019:1–10. 10.1136/bmjgh-2018-001257. 30997169 PMC6441291

[11] Marten R Mikkelsen B Shao R. Committing to implementation research for health systems to manage and control non-communicable diseases. Lancet Glob Health. 2021;9(2):e108–e109. 10.1016/S2214-109X(20)30485-X. 33357502 PMC7815625

[12] Kothari A McCutcheon C Graham ID. Defining integrated knowledge translation and moving forward: a response to recent commentaries. Int J Health Policy Manag. 2017; 6(5):299–300. 10.15171/ijhpm.2017.15. 28812820 PMC5417154

[13] Slattery P Saeri AK Bragge P. Research co-design in health: a rapid overview of reviews. 2020;9:1–13. 10.1186/s12961-020-0528-9. 32046728 PMC7014755

[14] Rudd BN Davis M Beidas RS. Integrating implementation science in clinical research to maximize public health impact: a call for the reporting and alignment of implementation strategy use with implementation outcomes in clinical research. Implement Sci. 2020;15(1):103. 10.1186/s13012-020-01060-5. 33239097 PMC7690013

[15] Sturke R Vorkoper S Bekker LG. Fostering successful and sustainable collaborations to advance implementation science: the adolescent HIV prevention and treatment implementation science alliance. J Int AIDS Soc. 2020;23 Suppl 5(Suppl 5):e25572. 10.1002/jia2.25572. 32869510 PMC7459159

[16] Partners. HEARD Project. Accessed March 8, 2022. https://www.heardproject.org/about-us/partners/

[17] Kilmarx PH Maitin T Adam T. A mechanism for reviewing investments in health research capacity strengthening in low-and middle-income countries. Ann Glob Health. 2020; 86(1):92. 10.5334/aogh.2941. 32832386 PMC7413164

[18] Yapa HM Bärnighausen T. Implementation science in resource-poor countries and communities. Implement Sci. 2018;13(1):154. 10.1186/s13012-018-0847-1. 30587195 PMC6307212

[19] Open access resources. Global Alliance for Chronic Diseases. Accessed February 28, 2022. https://www.gacd.org/research/researcher-resources

[20] Nachega JB Sam-Agudu NA Mellors JW Zumla A Mofenson LM. Scaling up Covid-19 vaccination in Africa–Lessons from the HIV pandemic. N Engl J Med. 2021;385(3):196–198. 10.1056/NEJMp2103313. 33789005 PMC8291168

[21] Zachariah R Rust S Berger SD. Building global capacity for conducting operational research using the SORT IT model: where and who? PLoS One. 2016;11(8):e0160837. 10.1371/journal.pone.0160837. 27505253 PMC4978462

