# Supplementary material for: Funders' Perspectives on Supporting Implementation Research in Low- and Middle-Income Countries
Source: Glob Health Sci Pract. 2022 Apr 28;10(2):e2100497. doi: 10.9745/GHSP-D-21-00497 (PMC9053148; doi:10.9745/GHSP-D-21-00497)
Supplement: GHSP-D-21-00497-supplement.pdf [file GHSP-D-21-00497-supplement.pdf]

Supplement to: Cardoso-Weinberg A, Alley C, Kupfer LE. Funders' perspectives on supporting implementation research in low-and - middle income countries. *Glob Health Sci Pract*. 2022;10(2): e2100497. https://doi.org/10.9745/GHSP-D-21-00497

Potential Limitations of Sample of Funders

The sample of funders may not be representative of all funders of implementation research in LMICs. Specifically, the study's lack of Asian donor agencies (due to a lack of referral during sampling), and its inclusion of only one Latin American agency, raises the potential limitation that the views captured from the interviews informed recommendations which may not be generalizable to all funders. However, it should be noted that UN funding agencies and programs such as EDCTP—based on partnerships—are not exclusively funded by high-income countries (here labeled as U.S. and European donors). They include financial and intellectual contributions from other regions, including low-and-middle-income countries. Moreover, these are highly collaborative programs and, through collaborative funding for R&D and implementation research, leverage funding and perspectives from other regions.

Some examples involving LMICs are:

Japan: https://www.ghitfund.org/newsroom/press/detail/302/

India, Serum Institute on vaccines development and access programs: https://www.eib.org/en/press/all/2020-226-eur-30-million-eib-backing-to-accelerate-tuberculosis-vaccine-development-in-africa

Argentina: http://www.edctp.org/news/mundo-sano-joins-forces-edctp-fund-research-neglected-infectious-diseases/

Supplement to: Cardoso-Weinberg A, Alley C, Kupfer LE. Funders' perspectives on supporting implementation research in low-and -middle income countries. *Glob Health Sci Pract*. 2022;10(2): e2100497. https://doi.org/10.9745/GHSP-D-21-00497

ESSENCE on Health Research

ESSENCE Implementation Science (IS)

Good Practice Document (GPD)

Interview Protocol

**PART I. Funding IS in LMICs**
I'd like to begin with some questions about your experience with funding IS in LMICs.
**1.1 & 1.2** ***Overview: Based on the information you provided in the survey, your organisation supports IS.***
1.1.1 How often does your organisation fund IS activities?
1.1.2 Have there been any changes over time in the way your organisation funds IS activities? (***prompt:*** e.g., has your organisation increased or decreased funding of IS activities, or has it changed its specifications and/or criteria for a Call for Proposals)?
1.2.1 Does your organisation fund projects that advance understanding of IS?
1.2.2 Does your organisation fund IS activities related to *specific interventions*?

**1.3 Arguments for funding IS and priority-setting**
1.3.1 Which proportion of your organisation's funding is allocated to IS in comparison to other research (***prompt:*** e.g., specific Calls for IS, or incorporating IS activities in other non-IS specific programmes)?
1.3.2 If possible, please provide the amount your organisation has awarded that directly relates to IS versus non-IS activities?
1.3.3 Can you describe the process your organisation follows to identify *priority areas* for funding, generally, and for IS in particular?

Supplement to: Cardoso-Weinberg A, Alley C, Kupfer LE. Funders' perspectives on supporting implementation research in low-and -middle income countries. *Glob Health Sci Pract*. 2022;10(2): e2100497. https://doi.org/10.9745/GHSP-D-21-00497

1.3.4 Can you suggest any case studies to demonstrate that funded IS activities have maximized the likelihood of research evidence uptake, or that have added value to intervention evaluation?
1.3.5 Can you recommend any specific examples that demonstrate how activities in other areas (i.e, areas not thought to be IS, per se) have been leveraged in ways that increased the adoption and/or implementation of evidence-based practices?
1.3.6 Are there any success stories and/or failures that you can mention related to IS activities funded by your organisation? (***ask respondents to send case studies by email***)

**1.4 Opportunities and challenges**
1.4.1 What would be some opportunities that additional funding of IS activities might bring to your organisation?
1.4.2 Are there potential benefits that have been identified and that led to the creation of a formalised procedure within your organisation to fund IS activities?
1.4.3 Are there any potential or current barriers to funding IS activities? Note, this might include barriers that exist outside your organisation.<br>- For example, is there competition across multiple funding streams?<br>- Does focus on IS pose any barrier (***prompt:*** e.g., no focus, lack of focus, or too narrow of focus)<br>- Are there structural barriers to funding IS activities (***prompt:*** e.g., your organisation cannot fund activities in specific LMICs or LMICs in general)?
1.4.4 If your organisation has any barriers to funding IS activities, has it addressed them? If so, how? (***prompt:*** For example, if funding activities in a LMIC is a barrier, has partnering with other organisations been needed/used as a strategy or solution?)
1.4.5 Are there opportunities for joint activities/partnering where common strategies may be agreed or might be mutually beneficial to funders?
1.4.6 Do you have any recommendations on best ways to fund IS in the future?

Supplement to: Cardoso-Weinberg A, Alley C, Kupfer LE. Funders' perspectives on supporting implementation research in low-and -middle income countries. *Glob Health Sci Pract*. 2022;10(2): e2100497. https://doi.org/10.9745/GHSP-D-21-00497

**1.5 Sustainability: Proposal Writing for IS Grants and Other Key Ingredients**
1.5.1 How can funders help ensure sustainability of interventions in LMICs?
1.5.2 Do you know of any IS projects and activities in which there are LMIC partners involved and engaged? Are any of these funded by LMICs?
1.5.3 Is there a specific funding mechanism (e.g., a Call) within your organisation to identify and support IS activities?
1.4.4 If so, how did you present these funding opportunities to the research community? Did you present this as an explicitly IS activity? (***Ask respondent to send separately in an email:*** please provide concrete examples such as the exact title and text of the Call for Proposals or an example of the process a grantee follows to be awarded a grant)
1.5.5 Agencies in LMICs: which ones (if any) are mandated by the government to invest in IS?
1.5.6 What importance, if any, do you place on LMICs' future ability to fund IS versus LMICs' continued reliance on support from funders in HICs? How can the ability of LMICs to fund their own IS activities be fostered?

Based on information you provided in the survey, your organisation does not yet support IS.
1.5.7 What are the main reasons why your organisation does not fund IS?
1.5.8 Why do you think your organisation should/should not fund IS?
1.5.9 What are the obstacles that funding of IS could bring to your organisation?
1.5.10 What opportunities could funding of IS bring to your organisation?

Supplement to: Cardoso-Weinberg A, Alley C, Kupfer LE. Funders' perspectives on supporting implementation research in low-and - middle income countries. *Glob Health Sci Pract*. 2022;10(2): e2100497. https://doi.org/10.9745/GHSP-D-21-00497

In case your organisation funds IS in the future:
1.5.11 Which activities do you think would be of interest? (geographical scope, population, diseases, etc.)
1.5.12 Which process do you think your organisation is likely to follow in order to award funding for IS? Would this be similar to the way other research areas are funded?
1.5.13 What do you think the proportion of IS funding would be compared to other areas?
1.5.14 Do you have any recommendations on best ways to fund IS in the future?
1.5.15 What would be the key ingredients of a good IS research proposal?

**PART II. Understanding IS and its Impact in LMICs**
Now I'd like to ask questions related to the understanding of IS and its impact in LMICs.
2.1.1 Is there a common understanding of IS within your organisation? Does your organisation have a working definition of IS?
2.1.2 How are your funding opportunities in IS presented and disseminated to their intended target audience?
**2.2** ***IS methodologies and frameworks (or theoretical perspectives) with focus on LMICs.***
2.2.1 Do any of the key weaknesses in research proposals you have received relate to their methodologies or frameworks/theoretical approaches? If so, how would you characterize these weaknesses?
2.2.2 What type of IS expertise is needed to ensure that the right types of methodologies and frameworks are included in proposals you receive?
**2.3** ***Potential impact that funded research has in LMICs.***
2.3.1 How much of your organisation's funded IS research focused on LMICs?

2.3.2 How important is impact of your investment to you as a funder and how do you measure it?
2.3.3 Can you give examples of any activities of which you are aware and that are intended to ensure sustainability of IS interventions in LMICs? Of the ones mentioned, which ones are funded by your organisation?
**2.4 *Current IS activities***
2.4.1 Do you have a sense that there are many research institutions with strengths in *Implementation Research*, or with capacity for conducting implementation studies? (Also ask respondent to send this information by email to help with mapping
2.4.1.a ***If yes:*** Can you suggest any examples of IS activities that you think would be useful to include in a Good Practice Document as a *case study*, especially those which may exemplify how IS influences policy and practice?
2.4.1.b ***If no:*** Can you suggest any the names of any individuals who might be able to provide information about current IS activities? Could you please provide their contact information?

**PART III. Building Capacity**
**3.1 *I'd like to now shift focus to questions related to building capacity aspects in IS research and training.***
3.1.1 When thinking about IS, what does "building capacity" mean to you?
3.1.2 Based on the information you provided in the survey, your organisation has (or has not) provided capacity building opportunities for IS.
**If the organisation previously reported that it *had not:***
3.1.3 Since answering the survey, has there been a change? Has your organisation provided capacity building opportunities for IS?
**If the organisation *has:***
3.1.4 Can you describe how your organisation has been providing capacity building opportunities for IS (ask respondents to send summaries of projects supported by email)

3.1.5 Do you think there is scope for capacity building in IS? Where would investments in capacity building for IS be best placed?
3.1.7 Can you provide any examples of how capacity building elements were successfully embedded/incorporated into IS funders' programmes ***(prompt:*** e.g., mentoring, advanced training opportunities)?
**3.2 *Learning and training for IS researchers.***
3.2.1 Are you aware of any learning and training opportunities for IS?
3.2.2 What form do these IS learning and training opportunities take? (***prompt:*** Are they initiatives? Certificate programs? Degree programs?)
3.2.3 What do the curricula of these training opportunities look like? (***prompt:*** What courses are offered/required? Any key differences among existing curricula?)
3.2.4 Where are these learning and training programs offered?
3.2.5 Are you aware of any training for IS that is offered in LMICs and/or by LMIC institutions?
3.2.6 Are you aware of any IS education training opportunities that are planning to be offered in the future?
3.2.7 Can you recommend any individuals who might be able to tell us more about learning and training opportunities for IS? Can you provide their contact information?

**PART IV: In Closing...**
**4.1 Before we conclude this interview, is there something about your experience (or lack of experience) in funding of IS that should be mentioned but that we have not yet had a chance to discuss?**
**4.2 Thank you for your participation. If you have any other thoughts you would like to share, please do not hesitate to email me at (*prompt:*** give email address**).**
